# Use of infrared thermography in the detection of superficial phlebitis in adult intensive care unit patients: A prospective single-center observational study

**DOI:** 10.1371/journal.pone.0213754

**Published:** 2019-03-13

**Authors:** Frank Doesburg, Joya M. Smit, Wolter Paans, Marisa Onrust, Maarten W. Nijsten, Willem Dieperink

**Affiliations:** 1 University of Groningen, University Medical Center Groningen, Department of Critical Care, Groningen, The Netherlands; 2 Hanze University of Applied Sciences, Groningen, The Netherlands; University Magna Graecia of Catanzaro, ITALY

## Abstract

Common methods to detect phlebitis may not be sufficient for patients in the intensive care unit (ICU). The goal of this study was to investigate the feasibility of infrared (IR) thermography to objectively detect phlebitis in adult ICU patients. We included a total of 128 adult ICU-patients in a pilot and subsequent validation study. Median [interquartile range] age was 62 [54–71] years and 88 (69%) patients were male. Severity of phlebitis was scored using the visual infusion phlebitis (VIP)-score, ranging from 0 (no phlebitis) to 5 (thrombophlebitis). The temperature difference (ΔT) between the insertion site and a proximal reference point was measured with IR thermography. In 78 (34%) catheters early phlebitis and onset of moderate phlebitis was observed (VIP-score of 1–3). In both the pilot and the validation study groups ΔT was significantly higher when the VIP-score was ≥1 compared to a VIP-score of 0 (p<0.01 and p<0.001, respectively). Multivariate analysis identified ΔT (p<0.001) and peripheral venous catheter (PVC) dwell time (p = 0.001) as significantly associated with phlebitis. IR thermography may be a promising technique to identify phlebitis in the ICU. An increased ΔT as determined with thermography may be a risk factor for phlebitis.

## Introduction

Phlebitis, i.e. inflammation of a superficial vein, is one of the most common complications associated with the use of peripheral venous catheters (PVCs) [1]. Phlebitis may occur in conjunction with the formation of a blood clot (thrombophlebitis) but the exact relation between phlebitis and thrombus formation is unknown [2,3]. As phlebitis may cause veins to become unfit for future intravenous access it is important that phlebitis is identified in a timely manner so that serious or lasting complications can be prevented [1,2,4].

To our knowledge, there is no generally accepted gold standard for the assessment of phlebitis, which is illustrated by the fact that there are over 70 different scoring systems for identifying and classifying the severity of PVC-related phlebitis [5]. The Visual Infusion Phlebitis (VIP) score is one of the most frequently used scoring systems for assessing phlebitis in the United States [6,7]. The VIP-score describes 6 levels of severity which all correspond to a number of visually identifiable signs, ranging from 0 (no phlebitis) to level 5 (thrombophlebitis) [8]. For example, when a patient experiences pain or when redness is observed at the insertion site this corresponds to a VIP-score of 1 (early onset phlebitis).

Patients in the intensive care unit (ICU) receive twice the amount of medication compared to patients on a regular ward [9]. As this often requires intravenous access, phlebitis may be more likely to occur in this group. However, the VIP-score is less suitable for use in the ICU as scoring pain is difficult in ICU patients who are often sedated or receive analgesics. Early signs of phlebitis, such as a VIP-score of 1, are therefore easily missed, which may lead to worsening of the phlebitis.

The VIP-score does not take an increased skin temperature around the insertion site into account, although this is considered to be a sign of phlebitis [10]. As an elevated temperature is likely to occur in conjunction with signs of inflammation such as redness and pain, this may facilitate the detection of phlebitis using infrared (IR) temperature measurements. An important advantage of IR thermography is that it does not require the skin surrounding the insertion site to be touched, which reduces the risk of contamination. The temperature at a nearby unaffected site could be used as a reference, and the temperature difference (ΔT) between the insertion site and nearby reference point may be indicative for the presence of phlebitis. IR thermography has already been applied in the detection of fever, deep venous thrombosis, peripheral circulatory impairment, breast cancer and malignant melanoma [11,12]. IR thermography may be an objective alternative to scoring systems that do not take skin temperature into account. However, the detection of superficial vein phlebitis with IR thermography has not been described before. Therefore, the objective of this study was to investigate the feasibility of IR thermography in phlebitis detection in adult ICU patients.

## Methods

This study was a prospective single-center observational study which consisted of a pilot and a subsequent validation study. Ethical approval was obtained from the Institutional Review Board of the University Medical Center Groningen, The Netherlands (METc.2016/119). Informed consent was waived as this was an observational study and study data were analyzed anonymously.

### Setting

This study was performed in a 42-bed level III ICU of a university hospital in The Netherlands. Nearly every patient admitted to the ICU has a PVC which is newly inserted in the emergency room, operating room or in the ICU in an aseptic way following hospital protocols. After hand disinfection with alcohol (Sterillium) the insertion site is cleaned using chlorhexidine 0.5% dissolved in 70% alcohol. The most suitable catheter and gauge is selected. Then, using non-sterile gloves the catheter is inserted by a trained nurse or physician. The catheter is then fixated using a transparent sterile dressing. The ICU nurses daily inspect the insertion site and the relevant extremity. They assess pain, extravasation, hematoma, irritation and infection. The sterile dressing is replaced when it no longer adheres to the skin, is damp or dirty and the infusion system (IV bags, lines and other disposable connectors) is replaced every 96 hours. Indications for PVC removal are phlebitis, subcutaneous administration, expiration of the indication, occlusion or leakage of the catheter.

### Patient selection

As this was a feasibility study, no formal sample size calculation was performed. Both the pilot and validation substudies included all adult ICU patients receiving IV treatment through one or more PVCs within their respective study periods. Patients below the age of 18 years, patients requiring immediate care (e.g. during resuscitation), patients in a strict isolation regime, patients refusing to be part of the study, and those on comfort measures were excluded.

### Data collection

The pilot study was conducted in the period from April 18^th^ until May 12^th^ of 2017 and the validation study was conducted from October 19^th^ until November 11^th^ of 2017. Data collection was performed in daily rounds including all eligible patients present in the ICU. Patients who met the inclusion criteria were assessed using a case report form (CRF), which incorporated known risk factors sex, PVC location, PVC dwell time, antibiotic administration, infusion fluids [7,13,14], and the VIP-scoring system. Two researchers independently assessed the insertion site and then discussed the VIP-score together to reach consensus on the grade of phlebitis. A full description of the VIP-scoring system is listed in S1 Table. For every PVC a separate IR measurement was performed and a separate CRF was filled in. When a patient had multiple PVCs at the same time, the order in which they were assessed was arbitrary. Temperature data from the IR images were downloaded from the camera and processed daily after data collection.

### Pilot study

The primary objective of the pilot study was to investigate the feasibility and requirements of using an IR camera in the detection of phlebitis. A Fluke TiX580 infrared camera (Fluke Europe B.V. Havé-Digitap, NL) was used for this purpose. This camera has a measurement range between -20 degrees Celsius (°C) and +800°C and a reported accuracy of ±2°C. In order to increase reliability of the IR measurements both the insertion site and a reference point approximately 10 cm proximal from the insertion site were captured in a single IR image (S1 Fig). We hypothesized that the occurrence of phlebitis would lead to a higher local skin temperature compared to the reference point. This difference in temperature in degrees Celsius (ΔT) was acquired from the infrared images using Fluke SmartView version 4.1 software.

### Validation study

The results of the pilot study indicated that statistically significant temperature differences were present in patients with and without signs of phlebitis. We therefore continued the study with protocol refinements. This included the use of an adapted measurement protocol, with an additional reference point and a more accurate IR camera.

In the validation study the reference points were measured to be 10cm from the insertion site, whereas in the pilot study this distance was estimated. A disposable ruler was placed on the extremity which was photographed. Depending on the location of the insertion site this rendered one or two reference points; one 10cm proximal and/or one 10cm distal the insertion site. This distal reference point was added because the proximal reference point could be affected by inflammation, thereby possibly reducing the reliability of the ΔT value. Both reference points and the insertion site were always captured within the same IR image to improve reliability. ΔT was measured between the insertion point of the PVC and the reference points on the same extremity.

In the validation study a FLIR T1030sc IR camera was used, which has a measurement range between at -40°C and +650°C and it an accuracy of ±1°C. Also, core body temperature as measured with a bladder probe was recorded from the patient monitor.

In both the pilot and the validation groups, demographics, reason of admission, and data on known risk factors for phlebitis, including BMI, immunologic deficiency, COPD, diabetes, and disease severity score APACHE-IV were recorded [14–16].

### Statistics

All data were analyzed with SPSS (IBM SPSS Statistics for Windows, Version 24.0 Armonk, IBM Corp.). For continuous variables, the mean with standard deviation (SD) is presented when normally distributed. In case of a skewed distribution, the median with interquartile range (IQR) is presented. Categorical variables are displayed as frequencies followed by percentages. Grouping was performed based on VIP-score (VIP-score = 0 vs. VIP-score ≥1). For assessing statistical significance the Student’s t-test, Mann Whitney U-test, chi-square test or Fisher exact test were used. The statistical significance was determined at a two-sided p-value of ≤0.05.

Backward stepwise multivariate logistic regression was performed for the combined pilot and validation study data and included variables that were described in literature as risk factors. Catheters inserted were the units of analysis. All variables that had a univariate relation with phlebitis with a p <0.10 were included in the stepwise multivariate analysis. At each step the variable with the largest p value was removed from the analysis until the multivariate model contained only variables that had a significant relation with phlebitis at a p <0.05.

## Results

Results for the pilot and validation study groups are presented separately. Baseline patient characteristics of the pilot and validation study groups are provided in Table 1. In the pilot study 103 unique PVCs were studied in 57 ICU patients and in the validation study 126 PVCs were studied in 71 ICU patients. Depending on the length of ICU stay some PVCs were monitored for multiple days. A total of 691 bedside measurements was performed. 66% of all unique insertion sites was rated a VIP-score of 0, 32% VIP 1 and 2% of VIP 2 or higher (S2 Table).

**Table 1:** Characteristics of patients in the pilot and validation study groups.

*Characteristics*	*Pilot study*	*Validation study*
**Number of patients**	57	71
Male, n (%)	35 (61)	53 (75)
Age, median [IQR]	65 [55–73]	61 [52–71]
**Admission diagnosis**		
Medical, n (%)	34 (60)	30 (42)
Surgical, n (%)	23 (40)	41 (58)
**Admission type**		
Elective, n (%)	20 (35)	23 (32)
Non-elective, n (%)	37 (65)	48 (68)
**Comorbidities**		
COPD, n (%)	8 (14)	13 (18)
Diabetes, n (%)	6 (10)	12 (17)
Immunologic deficiency, n (%)	5 (9)	7 (10)
**BMI**		
Underweight BMI <18.5, n (%)	2 (3)	0 (0)
Normal BMI 18.5–25, n (%)	26 (46)	37 (52)
Overweight BMI 25–30, n (%)	21 (37)	22 (31)
Obese BMI >30, n (%)	8 (14)	12 (17)
APACHE IV, median [IQR]	63 [48–78]	67 [54–86]
	n = 56	n = 68

Notes:

IQR: Interquartile range

COPD: Chronic Obstructive Pulmonary Disease

Immunologic deficiency: Patient received immunosuppressive therapy, corticosteroids, chemo- or radiotherapy, or has humoral or cellular deficiency

BMI: Body Mass Index

APACHE: Acute Physiology and Chronic Health Evaluation

IR temperature measurements for the pilot and validation studies are presented in Table 2. The absolute temperature at the insertion site as determined by infrared thermography was higher in patients with a VIP-score ≥1 compared to patients with a VIP-score of 0 in both groups (pilot, validation; p = 0.006, p<0.001). The ΔT was higher for patients with a VIP-score ≥1 compared to patients with a VIP-score of 0 in both groups (pilot, validation; p = 0.002, p<0.001). It must be noted that ΔT in both groups was calculated using the proximal reference point, as a distal reference point was not always available (e.g. when the insertion site was located in the hand or foot). The validation study showed that in particular the ΔT between the insertion site and proximal reference point showed a good discrimination between a VIP-score of 0 and ≥1.

**Table 2:** Infrared temperature measurements of the pilot and validation study groups in degrees Celsius.

*Characteristics*	*VIP-score 0*	*VIP-score ≥1*	*p*
**Pilot study group**	n = 79	n = 24	
Insertion site, median [IQR]	32.0 [29.8–33.8]	33.8 [32.4–35.5]	**0.006**^**3**^
Proximal reference point, median [IQR]	33.0 [31.6–35.0]	34.2 [32.8–35.1]	0.097^**3**^
ΔT^1^, median [IQR]	-0.90 [-2.10–0.10]	0.6 [-1.25–1.23]	**0.002**^**3**^
**Validation study group**	n = 72	n = 54	
Insertion site, median [IQR]	32.5 [31.0–34.5]	34.5 [33.2–35.8]	**<0.001**^**3**^
Proximal reference point, median [IQR]	33.4 [31.9–35.0]	34.5 [33.5–35.8]	**0.002**^**3**^
ΔT^1^, median [IQR]	-0.45 [-1.98–0.48]	0.45 [-0.30–0.90]	**<0.001**^**3**^
Distal reference point, median [IQR]	33.7 [31.7–35.3]	34.9 [32.7–36.0]	0.074^**3**^
	n = 47	n = 42	
ΔT^2^, median [IQR]	-0.40 [-1.40–0.70]	0.10 [-0.90–1.62]	0.129^**3**^
	n = 47	n = 42	

Notes:

P-values in bold font are statistically significant at a p <0.05 level

IQR: Interquartile range

SD: Standard deviation

^1^Temperature difference between insertion site and the proximal reference point on the same extremity

^2^Temperature difference between insertion site and the distal reference point on the same extremity

^3^Mann Whitney U test

PVC characteristics for the pilot and validation study groups are listed in S3 Table. Both in the pilot and in the validation study groups PVC dwell time was significantly higher in patients with a VIP-score ≥1 compared to VIP 0 (pilot study median [IQR] VIP 0 vs. VIP≥1; 21 [7.7–63.2] vs. 91.5 [29.5–258.7] hours, p <0.001. The validation study median [IQR] VIP 0 vs. VIP≥1; 28 [19.2–70.5] vs. 100.5 [52.2–172.0] hours, p <0.001). Body temperature had a linear relationship with both the temperature at the insertion site and the temperature at the proximal reference point (S4 Table).

Univariate and multivariate logistic regression analyses for the combined pilot and validation studies are displayed in Table 3. PVC dwell time and ΔT were both associated with phlebitis (p<0.001 for both variables).

**Table 3:** Univariate and multivariate analysis of phlebitis.

	Univariate analysis			Multivariate analysis		
Variable	Odds ratio	95% Confidence Interval	*p*	Odds ratio	95% Confidence Interval	*p*
Age	0.985	0.965–1.005	0.145			
Sex	0.691	0.372–1.285	0.243			
BMI	1.067	0.996–1.142	0.064			
PVC side	1.017	0.588–1.758	0.952			
PVC in foot	1.490	0.498–4.456	0.476			
PVC dwell time (hours)	1.007	1.003–1.010	<0.001	1.006	1.002–1009	0.001
ΔT (^o^C)	1.667	1.351–2.057	<0.001	1.494	1.202–1.855	<0.001
Elective admission	0.433	0.223–0.838	0.013			
Immunologic deficiency	0.666	0.267–1.665	0.385			
COPD	1.144	0.540–2.424	0.726			
Diabetes	1.279	0.597–2.741	0.527			
Antibiotic use	1.857	0.961–3.590	0.066			
Infusion fluids	1.215	0.305–4.835	0.782			
Admission diagnosis	1.323	0.752–2.323	0.331			
APACHE IV score	1.006	0.995–1.018	0.264			
				AUC: 0.76		
			

Notes:

BMI: Body mass index

PVC: Peripheral venous catheter

ΔT: Temperature difference between the insertion site and the proximal reference point on the same extremity

Immunologic deficiency: Patient received immunosuppressive therapy, corticosteroids, chemo- or radiotherapy, or has humoral or cellular deficiency

COPD: Chronic Obstructive Pulmonary Disease

APACHE: Acute Physiology and Chronic Health Evaluation

AUC: Area under the ROC curve using the multivariate function (with PVC and ΔT dwell time as independent variables) for predicting phlebitis as defined by VIP-score ≥1.

## Discussion

The objective of this study was to investigate the feasibility of IR thermography in phlebitis detection in adult ICU patients. A pilot study was conducted to identify the initial requirements for the practical application and the initial feasibility of IR thermography in the detection of phlebitis. The results of the pilot study were confirmed in a more formalized validation study.

The ΔT values were used to indicate the difference in temperature between unaffected skin and the catheter insertion site, as a single absolute temperature measurement might provide less information on the presence of a local inflammatory process. Patients with a higher ΔT were more likely to show early signs of phlebitis. Although the validation study used a more precise measurement of reference points, ΔT values measured between the insertion site and the proximal reference point were associated with phlebitis in both study groups. The fact that the ΔT values were relatively small overall may be partly explained by the relatively cold infusion fluid with a room temperature of approximately 20 degrees Celsius, reducing the temperature difference of the insertion site compared to the reference point. When a patient develops phlebitis, the local inflammatory response raises the temperature at the insertion site, being one of the classic signs of inflammation [17]. Hence the measured temperature at the insertion site will be higher than that of the reference point. These findings suggest that IR thermography may be useful to objectively identify phlebitis in early stages.

As far as we know, the detection of superficial vein phlebitis with IR thermography has not been described in literature before. Most medical applications of IR thermography focus on the detection of deep venous thrombosis, severe infection and non-invasive cancer detection [12,18–24]. These applications often examine thermal patterns of an affected limb and the interpretation is interpreter-dependent. In the current study numerical temperature values from the IR images were used instead, which is a more objective method. Hypo- or hyperthermia may have an influence on the IR thermography measurements as it may affect the patient’s skin temperature. Before it is used in clinical practice the impact of factors that influence IR measurements, such as hypo- and hyperthermia, cold infusion fluids and heat blankets, requires further investigation.

In our multivariate model we found that PVC dwell time was a risk factor for the occurrence of phlebitis, which is in agreement with previous studies [13,14,25–27]. Other risk factors described in literature, such as gender, age, antibiotics, BMI, chronic obstructive pulmonary disease (COPD), diabetes, immunologic deficiency or insertion site were not significant in this study [13,16]. Patients with COPD (who are often smokers) and diabetics may be more vulnerable to phlebitis [16,28]. Although statistical power probably is an issue with respect to our data, the lack of evidence for gender as a risk factor is congruent with Salgueiro-Oliveira et al. (2012).

34% of insertion sites were rated with a VIP-score of ≥1, which falls within the 20–40% range reported in earlier studies [1,13,29]. It is noteworthy that the current study was performed in the ICU whereas other studies were performed in general wards or surgical departments [13,16].The high level of care and relatively short length of stay in the ICU and consequently shorter duration that PVCs are in place may explain why mostly early stage phlebitis (VIP-score of 1) was observed.

Some limitations apply to our study. The researchers were not blinded and ICU nurses knew the goal of this observational study which may have led to increased vigilance for signs of phlebitis. Although all insertion sites were scored by two researchers, inter-rater agreement on the VIP-score was not recorded. The VIP-score was used for determining the grade of phlebitis at the insertion site. A limitation of the score we used is that it has not been validated for patients who are admitted to the ICU and who are often sedated and receive analgesics. This may influence the VIP-score because only slight pain can already result in a VIP-score of 1. Also, insertion sites in dark skinned people were more difficult to score with this subjective measuring instrument. In this study we did not correct for the temperature nor for the administration rate of the infusion fluid. Fast infusion of a cold fluid could make the temperature at the insertion site lower than that of the reference point, possibly leading to false negative results.

In a follow-up on this study we plan to track patients from ICU admission to discharge from the general ward. Prevalence and severity of phlebitis at the ward may be higher as nurses at the ward typically have less time to manage infusion insertion sites than ICU nurses. Also, comparing the level of agreement between IR thermography and the VIP-score could provide insight in the accuracy of IR thermography in the detection of the various grades of phlebitis. A comparison of IR thermography and the VIP-score with a third measurement, e.g. ultrasonography (which is widely used to assess the condition of the veins) may reveal whether there are performance differences between IR thermography and the VIP-score in the detection of phlebitis [30]. Although the quality/price ratio has improved for IR cameras, higher accuracy and speed are generally related to a higher price. Considering IR measurements only take a minute to perform and require minimal instructions for use, this may ease the introduction of IR thermography in the ICU.

## Conclusion

This study shows that infrared thermography may be a promising and helpful technique to objectively identify the early development of phlebitis in the ICU. An increased ΔT appears to be a risk factor for phlebitis.

## Supporting information

S1 Fig Infrared image of a patient with a VIP 1 score. The maximum temperature at the insertion site (left circle) is 36.4°C and that of the proximal reference point (right circle) is 34.4°C. The ΔT in this case is 2.0°C.
(TIF)

S1 Table The VIP scoring system. (DOCX)

S2 Table Frequency distribution of VIP-scores in the pilot and validation study groups. (DOCX)

S3 Table Peripheral venous catheter characteristics of the pilot and validation study groups. (DOCX)

S4 Table Univariate analysis of body temperature as a predictor for infrared thermography measurements. (DOCX)

S1 File Anonymized study data file in SPSS format. (SAV)

## Abbreviations

BMI: Body mass index
CRF: Case report form
COPD: Chronic Obstructive Pulmonary Disease
Delta T; ΔT: Temperature difference between an insertion site and a nearby proximal reference point on the same extremity
ICU: Intensive care unit
IV: Intravenous
IR: Infrared
IQR: Interquartile range
PVC: Peripheral venous catheter
VIP-score: Visual Infusion Phlebitis-score
