# Supplementary figures and images for: Use of infrared thermography in the detection of superficial phlebitis in adult intensive care unit patients: A prospective single-center observational study
Source: PLoS One. 2019 Mar 13;14(3):e0213754. doi: 10.1371/journal.pone.0213754 (PMC6415825; doi:10.1371/journal.pone.0213754)

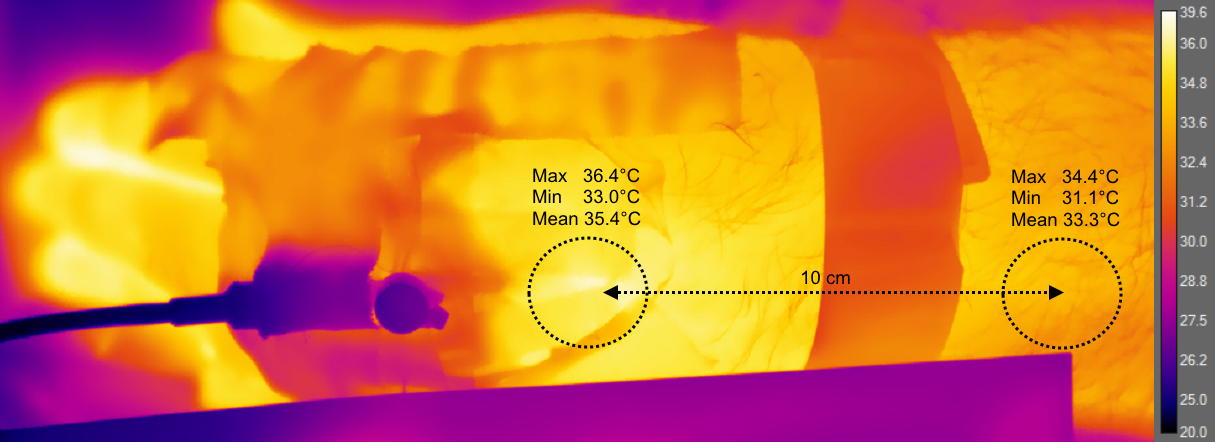

Supplement: S1 Fig — The maximum temperature at the insertion site (left circle) is 36.4°C and that of the proximal reference point (right circle) is 34.4°C. The ΔT in this case is 2.0°C. (TIF) [file pone.0213754.s001.tif]
