# Supplementary material for: Use of infrared thermography in the detection of superficial phlebitis in adult intensive care unit patients: A prospective single-center observational study
Source: PLoS One. 2019 Mar 13;14(3):e0213754. doi: 10.1371/journal.pone.0213754 (PMC6415825; doi:10.1371/journal.pone.0213754)
Supplement: S1 Table — (DOCX) [file pone.0213754.s002.docx]

**S1 Table. The VIP scoring system.**

| ***VIP level*** | ***Symptoms*** | ***Interpretation and action*** |
| --- | --- | --- |
| 0 | IV site appears healthy | No signs of phlebitis  Observe cannula |
| 1 | One of the following signs are evident:   - Slight pain at IV site - Redness near IV site | Possible first sign of phlebitis  Observe cannula |
| 2 | Two of the following signs are evident:   - Pain - Erythema - Swelling | Early stage of phlebitis  Resite the cannula |
| 3 | All of the following signs are evident:   - Pain along the path of the cannula - Erythema - Induration | Medium stage of phlebitis  Resite the cannula  Consider treatment |
| 4 | All of the following signs are evident and extensive:   - Pain along the path of the cannula - Erythema - Induration - Palpable venous cord | Advanced stage of phlebitis and start of thrombophlebitis  Resite the cannula  Consider treatment |
| 5 | All of the following signs are evident and extensive:   - Pain along the path of the cannula - Erythema - Induration - Palpable venous cord - Pyrexia | Advanced stage of phlebitis and start of thrombophlebitis  Resite the cannula  Initiate treatment |
