# Supplementary material for: Use of infrared thermography in the detection of superficial phlebitis in adult intensive care unit patients: A prospective single-center observational study
Source: PLoS One. 2019 Mar 13;14(3):e0213754. doi: 10.1371/journal.pone.0213754 (PMC6415825; doi:10.1371/journal.pone.0213754)
Supplement: S2 Table — (DOCX) [file pone.0213754.s003.docx]

S2 Table. Frequency distribution of VIP scores in the pilot and validation study groups.

| ***VIP score*** | ***Pilot***  ***study***  **n (%)** | ***Validation study***  **n (%)** | ***Total***  ***n (%)*** |
| --- | --- | --- | --- |
| ***0*** | 79 (77) | 72 (57) | 152 (66) |
| **1** | 24 (23) | 49 (39) | 73 (32) |
| **2** | 0 (0) | 3 (2) | 3 (1) |
| **3** | 0 (0) | 2 (2) | 2 (1) |
| **Total** | 103 (100) | 126 (100) | 229 (100) |
