# Supplementary material for: Use of infrared thermography in the detection of superficial phlebitis in adult intensive care unit patients: A prospective single-center observational study
Source: PLoS One. 2019 Mar 13;14(3):e0213754. doi: 10.1371/journal.pone.0213754 (PMC6415825; doi:10.1371/journal.pone.0213754)
Supplement: S3 Table — (DOCX) [file pone.0213754.s004.docx]

S3 Table. Peripheral venous catheter characteristics of the pilot and validation study groups.

|  | **Pilot study** |  |  | **Validation study** |  |  |
| --- | --- | --- | --- | --- | --- | --- |
| **Characteristics** | **VIP-score 0** | **VIP-score ≥1** | **p** | **VIP-score 0** | **VIP-score ≥1** | **p** |
|  | **n=79** | **n=24** |  | **n=72** | **n=54** |  |
| **PVC in Gauge*** |  |  |  |  |  |  |
| 14, n (%) | 9 (11.5) | 4 (17) | n.s.^1^ | 11 (15) | 5 (9) | n.s.^1^ |
| 16, n (%) | 9 (11.5) | 6 (25) |  | 12 (17) | 8 (15) |  |
| 18, n (%) | 18 (23) | 6 (25) |  | 14 (19) | 18 (33) |  |
| 20, n (%) | 39 (50) | 8 (33) |  | 34 (47) | 20 (37) |  |
| 22, n (%) | 3 (4) | 0 (0) |  | 1 (1) | 3 (6) |  |
|  |  |  |  |  |  |  |
| PVC dwell time in hours, median [IQR]* | 32 [15 - 66] | 91 [29 - 259] | 0.002^2^ | 28 [19 - 70] | 100 [52 - 172] | <0.001^2^ |
| **Placement** |  |  |  |  |  |  |
| Right, n (%) | 34 (43) | 12 (50) | n.s.^1^ | 37 (51) | 25 (46) | n.s.^1^ |
| Left, n (%) | 45 (57) | 12 (50) |  | 35 (49) | 29 (54) |  |
| **Anatomical insertion site** |  |  |  |  |  |  |
| Hand | 36 (46) | 4 (17) | n.s.^1^ | 30 (42) | 20 (37) | n.s.^1^ |
| Wrist | 0 (0) | 0 (0) |  | 10 (14) | 3 (6) |  |
| Forearm (dorsal) | 28 (35) | 9 (37) |  | 10 (14) | 7 (13) |  |
| Forearm (ventral) | 3 (4) | 4 (17) |  | 9 (12) | 9 (17) |  |
| Elbow | 7 (9) | 5 (21) |  | 8 (11) | 11 (20) |  |
| Upper arm (dorsal) | 1 (1) | 0 (0) |  | 0 (0) | 0 (0) |  |
| Upper arm (ventral) | 1 (1) | 0 (0) |  | 0 (0) | 0 (0) |  |
| Foot | 3 (4) | 2 (8) |  | 5 (7) | 4 (7) |  |
| **Intravenous antibiotics** |  |  |  |  |  |  |
| Yes, n (%) | 15 (19) | 5 (21) | n.s.^1^ | 10 (14) | 16 (30) | 0.031^1^ |
| No, n (%) | 64 (81) | 19 (79) |  | 62 (86) | 38 (70) |  |
|  |  |  |  |  |  |  |
| ^1^chi-square test  ^2^Mann Whitney U test  *n= 78 in the VIP-score 0 group due to missing data in hospital database  IQR: interquartile range  n.s: not significant | | | | | | |
