# Supplementary material for: Use of infrared thermography in the detection of superficial phlebitis in adult intensive care unit patients: A prospective single-center observational study
Source: PLoS One. 2019 Mar 13;14(3):e0213754. doi: 10.1371/journal.pone.0213754 (PMC6415825; doi:10.1371/journal.pone.0213754)
Supplement: S4 Table — (DOCX) [file pone.0213754.s005.docx]

S4 Table. Univariate analysis of body temperature as a predictor for infrared thermography measurements.

| **Independent variable** | **Dependent variable** | **B (95% Confidence Interval)** | ***p*** |
| --- | --- | --- | --- |
| Body temperature | Temperature at insertion site (^o^C) | 0.91 (0.37 –1.46) | 0.001 |
|  | Temperature at distal reference point (^o^C) | 0.043 (-0.020 – 0.105) | 0.177 |
|  | Temperature at proximal reference point (^o^C) | 0.096 (0.029 – 0.164) | 0.005 |
|  | ΔT^1^  (^o^C) | -0.009(-0.096 – 0.077) | 0.831 |
|  | ΔT^2^  (^o^C) | 0.076 (-0.016 – 0.168) | 0.105 |

Notes:

^1^Temperature difference between the insertion site and the distal reference point on the same extremity

^2^Temperature difference between the insertion site and the proximal reference point on the same extremity
